# 4-Benzyl­sulfanyl-1*H*-pyrazolo­[3,4-*d*]pyrimidine

**DOI:** 10.1107/S160053681302789X

**Published:** 2013-10-16

**Authors:** Mohammed El Fal, Youssef Ramli, El Mokhtar Essassi, Mohamed Saadi, Lahcen El Ammari

**Affiliations:** a Laboratoire de Chimie Organique Hétérocyclique, URAC 21, Pôle de Compétences Pharmacochimie, Université Mohammed V-Agdal, BP 1014 Avenue Ibn Batouta, Rabat, Morocco; b Laboratoire National de Contrôle des Médicaments, D M P, Ministère de la Santé, Madinat Al Irnane, BP 6206, Rabat, Morocco; c Laboratoire de Chimie du Solide Appliquée, Faculté des Sciences, Université Mohammed V-Agdal, Avenue Ibn Battouta, BP 1014, Rabat, Morocco

## Abstract

The pyrazolo­[3,4-*d*]pyrimidine ring system of the title compound, C_12_H_10_N_4_S, is essentially planar [maximum deviation = 0.025 (1) Å for the C atom bearing the S atom] and almost perpendicular to the phenyl ring [dihedral angle = 71.42 (6)°]. In the crystal, mol­ecules are linked *via* pairs of N—H⋯N hydrogen bonds, forming inversion dimers.

## Related literature

For the biological properties of pyrazolo­[3,4-*d*]pyrimidine derivatives, see: Rashad *et al.* (2008 ▶, 2011 ▶); Ballell *et al.* (2007 ▶). For related compounds, see: Moussaif *et al.* (2010 ▶); Ouzidan *et al.* (2011 ▶); Alsubari *et al.* (2011 ▶).
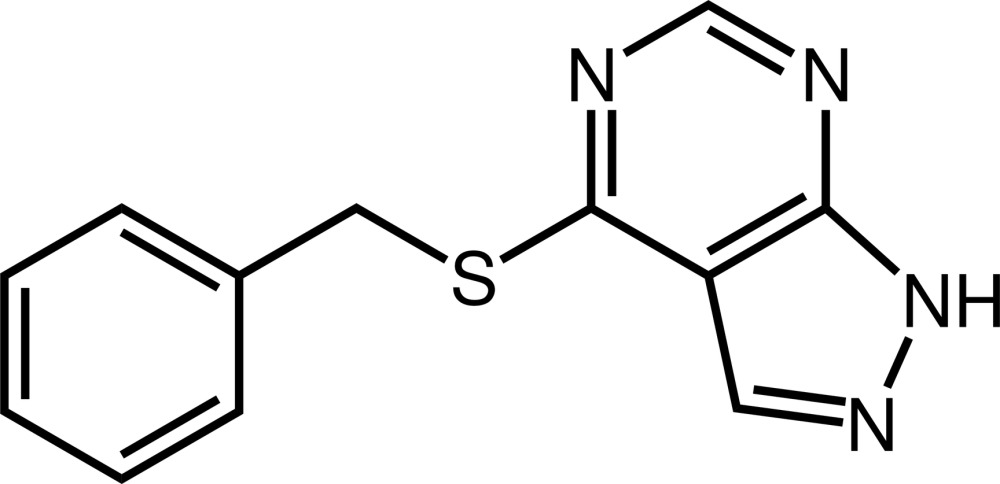


## Experimental

### 

#### Crystal data

C_12_H_10_N_4_S
*M* *_r_* = 242.30
Monoclinic, 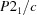

*a* = 9.4737 (3) Å
*b* = 5.1709 (2) Å
*c* = 23.6159 (8) Å
β = 96.823 (1)°
*V* = 1148.69 (7) Å^3^
*Z* = 4
Mo *K*α radiation
μ = 0.26 mm^−1^
*T* = 296 K
0.42 × 0.29 × 0.17 mm

#### Data collection

Bruker X8 APEXII diffractometer
Absorption correction: multi-scan (*SADABS*; Bruker, 2009 ▶) *T*_min_ = 0.960, *T*_max_ = 0.991
15333 measured reflections
3509 independent reflections
2885 reflections with *I* > 2σ(*I*)
*R*_int_ = 0.029

#### Refinement

*R*[*F* ^2^ > 2σ(*F* ^2^)] = 0.041
*wR*(*F* ^2^) = 0.112
*S* = 1.03
3509 reflections
154 parameters
H-atom parameters constrained
Δρ_max_ = 0.37 e Å^−3^
Δρ_min_ = −0.23 e Å^−3^

### 

Data collection: *APEX2* (Bruker, 2009 ▶); cell refinement: *SAINT* (Bruker, 2009 ▶); data reduction: *SAINT*; program(s) used to solve structure: *SHELXS97* (Sheldrick, 2008 ▶); program(s) used to refine structure: *SHELXL97* (Sheldrick, 2008 ▶); molecular graphics: *ORTEP-3 for Windows* (Farrugia, 2012 ▶); software used to prepare material for publication: *PLATON* (Spek, 2009 ▶) and *publCIF* (Westrip, 2010 ▶).

## Supplementary Material

Crystal structure: contains datablock(s) I. DOI: 10.1107/S160053681302789X/rz5086sup1.cif

Structure factors: contains datablock(s) I. DOI: 10.1107/S160053681302789X/rz5086Isup2.hkl

Supplementary material file. DOI: 10.1107/S160053681302789X/rz5086Isup3.cml

Additional supplementary materials:  crystallographic information; 3D view; checkCIF report

## Figures and Tables

**Table 1:** Hydrogen-bond geometry (Å, °)

*D*—H⋯*A*	*D*—H	H⋯*A*	*D*⋯*A*	*D*—H⋯*A*
N3—H3*N*⋯N2^i^	0.86	2.10	2.9429 (16)	168

Symmetry code: (i) 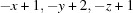.

**Fig. 1.**
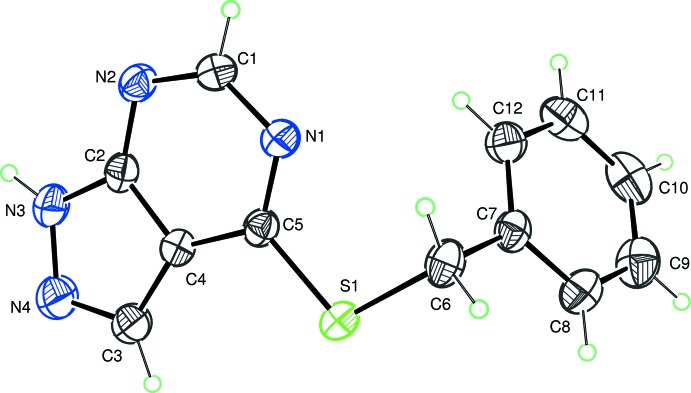
: Molecular plot the title compound with displacement ellipsoids drawn at the 50% probability level. H atoms are represented as small circles.

**Fig. 2.**
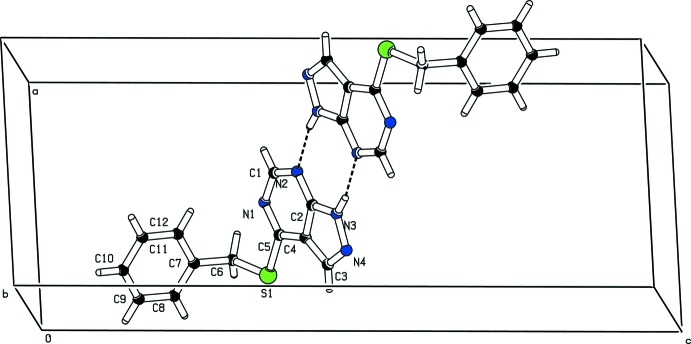
: Packing diagram of the title compound showing the linkage between centrosymmetrically related molecules by N3—H3N···N2 hydrogen bonds (dashed lines).

## supplementary crystallographic information

### 1. Comment

Pyrazolo[3,4-*d*]pyrimidine derivatives are an important class of heterocyclic pharmaceuticals because of their significant and broad spectrum of biological properties, antiviral (Rashad *et al.*, 2008); anti-mycobacterial (Ballell *et al.*, 2007) and anticancer (Rashad *et al.*, 2011). The present work is a continuation of the investigation of the sulfonamide derivatives published recently by our team (Moussaif *et al.*, 2010; Ouzidan *et al.*, 2011; Alsubari *et al.*, 2011).

The crystal structure of title compound is build up from two fused six-membered rings (N1 to N4 C1 to C5) linked to a benzylsulfanyl group (S1 C6 to C12) as shown in Fig. 1. The fused rings system is almost planar with the largest deviation from the mean plane being -0.025 (1) A° at C5 atom. The dihedral angle between the benzyl cycle (C7 to C12) and the mean plane through the pyrazolo[3,4-*d*]pyrimidine system is of 71.42 (6)°. In the crystal, each molecule is linked to its symmetry equivalent created by a crystallographic inversion center by pairs of N3–H3N···N2 hydrogen bonds, forming inversion dimers as shown in Fig. 2 and Table 1.

### 2. Experimental

1H,5*H*-Pyrazolo[3,4-*d*]pyrimidine-4-thione (0.5 g, 3.29 mmol), benzylchloride (0.6 ml, 6.8 mmol) and potassium carbonate (0.94 g, 6.8 mmol) with a catalytic amount of tetra-n-butylammonium bromide were stirred in DMF (15 ml) for 72 h. The solid material was removed by filtration and the solvent evaporated under vacuum. The solid product was purified by recrystallization from ethanol to afford yellow crystals in 60% yield.

### 3. Refinement

All H atoms could be located in a difference Fourier map. However, they were placed in calculated positions with C—H = 0.93-0.96 Å, N—H = 0.88 Å, and refined as riding on their parent atoms with *U*_iso_(H) = 1.2 *U*_eq_(C, N). In the last cycles of refinement, two outliers (0 0 2, 1 0 0) were omitted.

### Crystal data

**Table:**

C_12_H_10_N_4_S	*F*(000) = 504
*M**_r_* = 242.30	*D*_x_ = 1.401 Mg m^−^^3^
Monoclinic, *P*2_1_/*c*	Mo *K*α radiation, λ = 0.71073 Å
Hall symbol: -P 2ybc	Cell parameters from 3509 reflections
*a* = 9.4737 (3) Å	θ = 2.6–30.5°
*b* = 5.1709 (2) Å	µ = 0.26 mm^−^^1^
*c* = 23.6159 (8) Å	*T* = 296 K
β = 96.823 (1)°	Sheet, yellow
*V* = 1148.69 (7) Å^3^	0.42 × 0.29 × 0.17 mm
*Z* = 4	

### Data collection

**Table:**

Bruker X8 APEXII diffractometer	3509 independent reflections
Radiation source: fine-focus sealed tube	2885 reflections with *I* > 2σ(*I*)
Graphite monochromator	*R*_int_ = 0.029
φ and ω scans	θ_max_ = 30.5°, θ_min_ = 2.6°
Absorption correction: multi-scan (*SADABS*; Bruker, 2009)	*h* = −13→12
*T*_min_ = 0.960, *T*_max_ = 0.991	*k* = −7→7
15333 measured reflections	*l* = −33→33

### Refinement

**Table:**

Refinement on *F*^2^	Primary atom site location: structure-invariant direct methods
Least-squares matrix: full	Secondary atom site location: difference Fourier map
*R*[*F*^2^ > 2σ(*F*^2^)] = 0.041	Hydrogen site location: inferred from neighbouring sites
*wR*(*F*^2^) = 0.112	H-atom parameters constrained
*S* = 1.03	*w* = 1/[σ^2^(*F*_o_^2^) + (0.0573*P*)^2^ + 0.2818*P*] where *P* = (*F*_o_^2^ + 2*F*_c_^2^)/3
3509 reflections	(Δ/σ)_max_ = 0.001
154 parameters	Δρ_max_ = 0.37 e Å^−^^3^
0 restraints	Δρ_min_ = −0.23 e Å^−^^3^

### Special details

**Table:**

Geometry. All s.u.'s (except the s.u. in the dihedral angle between two l.s. planes) are estimated using the full covariance matrix. The cell s.u.'s are taken into account individually in the estimation of s.u.'s in distances, angles and torsion angles; correlations between s.u.'s in cell parameters are only used when they are defined by crystal symmetry. An approximate (isotropic) treatment of cell s.u.'s is used for estimating s.u.'s involving l.s. planes.
Refinement. Refinement of *F*^2^ against all reflections. The weighted *R*-factor *wR* and goodness of fit *S* are based on *F*^2^, conventional *R*-factors *R* are based on *F*, with *F* set to zero for negative *F*^2^. The threshold expression of *F*^2^ > σ(*F*^2^) is used only for calculating *R*-factors(gt) *etc*. and is not relevant to the choice of reflections for refinement. *R*-factors based on *F*^2^ are statistically about twice as large as those based on *F*, and *R*- factors based on all data will be even larger.

### Fractional atomic coordinates and isotropic or equivalent isotropic displacement parameters (Å^2^)

**Table:**

	*x*	*y*	*z*	*U*_iso_*/*U*_eq_	
C1	0.53146 (14)	0.5891 (3)	0.39990 (6)	0.0400 (3)	
H1	0.6186	0.6093	0.3860	0.048*	
C2	0.38176 (14)	0.7002 (2)	0.46100 (5)	0.0347 (3)	
C3	0.17220 (16)	0.5324 (3)	0.47363 (7)	0.0510 (4)	
H3	0.0914	0.4289	0.4698	0.061*	
C4	0.28477 (13)	0.5093 (2)	0.43999 (5)	0.0349 (3)	
C5	0.32168 (12)	0.3638 (2)	0.39401 (5)	0.0315 (2)	
C6	0.28394 (15)	0.0099 (3)	0.30385 (6)	0.0416 (3)	
H6A	0.3863	0.0157	0.3134	0.050*	
H6B	0.2568	−0.1699	0.2979	0.050*	
C7	0.24460 (14)	0.1560 (2)	0.24936 (6)	0.0369 (3)	
C8	0.12518 (16)	0.0842 (3)	0.21266 (6)	0.0467 (3)	
H8	0.0701	−0.0551	0.2219	0.056*	
C9	0.08797 (17)	0.2192 (4)	0.16251 (7)	0.0555 (4)	
H9	0.0086	0.1683	0.1380	0.067*	
C10	0.16673 (19)	0.4269 (4)	0.14851 (7)	0.0566 (4)	
H10	0.1402	0.5183	0.1150	0.068*	
C11	0.28603 (18)	0.4997 (3)	0.18457 (7)	0.0534 (4)	
H11	0.3401	0.6400	0.1752	0.064*	
C12	0.32523 (16)	0.3646 (3)	0.23458 (6)	0.0450 (3)	
H12	0.4060	0.4136	0.2584	0.054*	
N1	0.44627 (11)	0.4023 (2)	0.37454 (5)	0.0371 (2)	
N2	0.50762 (11)	0.7469 (2)	0.44184 (5)	0.0385 (2)	
N3	0.32520 (13)	0.8224 (2)	0.50327 (5)	0.0456 (3)	
H3N	0.3651	0.9487	0.5227	0.055*	
N4	0.19642 (15)	0.7201 (3)	0.51134 (6)	0.0583 (4)	
S1	0.20019 (4)	0.13646 (7)	0.363530 (14)	0.04065 (11)	

### Atomic displacement parameters (Å^2^)

**Table:**

	*U*^11^	*U*^22^	*U*^33^	*U*^12^	*U*^13^	*U*^23^
C1	0.0380 (6)	0.0415 (7)	0.0407 (7)	−0.0091 (5)	0.0060 (5)	−0.0020 (5)
C2	0.0405 (6)	0.0316 (6)	0.0305 (5)	−0.0006 (5)	−0.0017 (4)	−0.0004 (4)
C3	0.0425 (7)	0.0646 (10)	0.0478 (8)	−0.0093 (7)	0.0126 (6)	−0.0170 (7)
C4	0.0351 (6)	0.0369 (6)	0.0321 (6)	−0.0032 (5)	0.0020 (4)	−0.0023 (5)
C5	0.0335 (6)	0.0297 (5)	0.0301 (5)	−0.0011 (4)	−0.0007 (4)	0.0009 (4)
C6	0.0476 (7)	0.0327 (6)	0.0432 (7)	0.0040 (5)	−0.0008 (5)	−0.0070 (5)
C7	0.0396 (6)	0.0322 (6)	0.0388 (6)	0.0053 (5)	0.0044 (5)	−0.0080 (5)
C8	0.0434 (7)	0.0441 (7)	0.0506 (8)	0.0005 (6)	−0.0025 (6)	−0.0039 (6)
C9	0.0476 (8)	0.0655 (10)	0.0505 (8)	0.0123 (8)	−0.0060 (6)	−0.0025 (8)
C10	0.0608 (9)	0.0641 (10)	0.0464 (8)	0.0263 (8)	0.0126 (7)	0.0104 (7)
C11	0.0598 (9)	0.0468 (8)	0.0576 (9)	0.0059 (7)	0.0231 (7)	0.0055 (7)
C12	0.0445 (7)	0.0435 (8)	0.0480 (8)	−0.0009 (6)	0.0091 (6)	−0.0068 (6)
N1	0.0368 (5)	0.0373 (6)	0.0373 (5)	−0.0044 (4)	0.0050 (4)	−0.0037 (4)
N2	0.0421 (6)	0.0349 (6)	0.0377 (5)	−0.0080 (5)	0.0008 (4)	−0.0010 (4)
N3	0.0506 (7)	0.0457 (6)	0.0401 (6)	−0.0053 (5)	0.0040 (5)	−0.0130 (5)
N4	0.0521 (7)	0.0715 (9)	0.0535 (8)	−0.0077 (7)	0.0158 (6)	−0.0225 (7)
S1	0.03936 (18)	0.0419 (2)	0.04027 (18)	−0.01091 (13)	0.00279 (12)	−0.00697 (13)

### Geometric parameters (Å, º)

**Table:**

C1—N2	1.3234 (17)	C6—H6B	0.9700
C1—N1	1.3521 (17)	C7—C12	1.390 (2)
C1—H1	0.9300	C7—C8	1.3914 (19)
C2—N3	1.3455 (17)	C8—C9	1.384 (2)
C2—N2	1.3464 (17)	C8—H8	0.9300
C2—C4	1.3988 (17)	C9—C10	1.371 (3)
C3—N4	1.319 (2)	C9—H9	0.9300
C3—C4	1.4085 (19)	C10—C11	1.384 (3)
C3—H3	0.9300	C10—H10	0.9300
C4—C5	1.3992 (17)	C11—C12	1.385 (2)
C5—N1	1.3316 (16)	C11—H11	0.9300
C5—S1	1.7399 (12)	C12—H12	0.9300
C6—C7	1.5008 (19)	N3—N4	1.3637 (18)
C6—S1	1.8193 (14)	N3—H3N	0.8600
C6—H6A	0.9700		
			
N2—C1—N1	128.77 (12)	C8—C7—C6	120.02 (13)
N2—C1—H1	115.6	C9—C8—C7	120.27 (15)
N1—C1—H1	115.6	C9—C8—H8	119.9
N3—C2—N2	127.71 (12)	C7—C8—H8	119.9
N3—C2—C4	106.97 (12)	C10—C9—C8	120.74 (15)
N2—C2—C4	125.32 (12)	C10—C9—H9	119.6
N4—C3—C4	111.15 (13)	C8—C9—H9	119.6
N4—C3—H3	124.4	C9—C10—C11	119.54 (15)
C4—C3—H3	124.4	C9—C10—H10	120.2
C2—C4—C5	116.14 (11)	C11—C10—H10	120.2
C2—C4—C3	104.44 (11)	C10—C11—C12	120.27 (16)
C5—C4—C3	139.39 (12)	C10—C11—H11	119.9
N1—C5—C4	120.07 (11)	C12—C11—H11	119.9
N1—C5—S1	121.88 (9)	C11—C12—C7	120.41 (14)
C4—C5—S1	118.05 (9)	C11—C12—H12	119.8
C7—C6—S1	113.35 (9)	C7—C12—H12	119.8
C7—C6—H6A	108.9	C5—N1—C1	117.44 (11)
S1—C6—H6A	108.9	C1—N2—C2	112.17 (11)
C7—C6—H6B	108.9	C2—N3—N4	111.31 (12)
S1—C6—H6B	108.9	C2—N3—H3N	124.3
H6A—C6—H6B	107.7	N4—N3—H3N	124.3
C12—C7—C8	118.76 (14)	C3—N4—N3	106.12 (12)
C12—C7—C6	121.21 (12)	C5—S1—C6	103.63 (6)
			
N3—C2—C4—C5	178.01 (11)	C10—C11—C12—C7	−0.6 (2)
N2—C2—C4—C5	−2.39 (19)	C8—C7—C12—C11	0.8 (2)
N3—C2—C4—C3	−0.53 (15)	C6—C7—C12—C11	−178.96 (13)
N2—C2—C4—C3	179.08 (13)	C4—C5—N1—C1	−1.62 (18)
N4—C3—C4—C2	0.49 (18)	S1—C5—N1—C1	177.92 (10)
N4—C3—C4—C5	−177.49 (16)	N2—C1—N1—C5	−1.3 (2)
C2—C4—C5—N1	3.22 (17)	N1—C1—N2—C2	2.1 (2)
C3—C4—C5—N1	−178.96 (17)	N3—C2—N2—C1	179.40 (13)
C2—C4—C5—S1	−176.34 (9)	C4—C2—N2—C1	−0.13 (18)
C3—C4—C5—S1	1.5 (2)	N2—C2—N3—N4	−179.18 (13)
S1—C6—C7—C12	90.97 (14)	C4—C2—N3—N4	0.42 (16)
S1—C6—C7—C8	−88.78 (14)	C4—C3—N4—N3	−0.2 (2)
C12—C7—C8—C9	−0.1 (2)	C2—N3—N4—C3	−0.11 (19)
C6—C7—C8—C9	179.65 (13)	N1—C5—S1—C6	−3.06 (12)
C7—C8—C9—C10	−0.8 (2)	C4—C5—S1—C6	176.49 (10)
C8—C9—C10—C11	1.0 (2)	C7—C6—S1—C5	−90.38 (11)
C9—C10—C11—C12	−0.3 (2)		

### Hydrogen-bond geometry (Å, º)

**Table:**

*D*—H···*A*	*D*—H	H···*A*	*D*···*A*	*D*—H···*A*
N3—H3*N*···N2^i^	0.86	2.10	2.9429 (16)	168

Symmetry code: (i) −*x*+1, −*y*+2, −*z*+1.
